# Proliferating mesodermal cells in murine embryos exhibiting macrophage and lymphendothelial characteristics

**DOI:** 10.1186/1471-213X-8-43

**Published:** 2008-04-22

**Authors:** Kerstin Buttler, Taichi Ezaki, Jörg Wilting

**Affiliations:** 1 Centre of Anatomy, Department of Anatomy and Cell Biology, University Medicine Goettingen, Goettingen, Germany; 2 Department of Anatomy and Developmental Biology, Tokyo Women's Medical University, Tokyo, Japan; 3 University Medicine Goettingen, Centre of Anatomy, Department of Anatomy and Cell Biology, Kreuzbergring 36, 37075 Goettingen, Germany

## Abstract

**Background:**

The data on the embryonic origin of lymphatic endothelial cells (LECs) from either deep embryonic veins or mesenchymal (or circulating) lymphangioblasts presently available remain inconsistent. In various vertebrates, markers for LECs are first expressed in specific segments of embryonic veins arguing for a venous origin of lymph vessels. Very recently, studies on the mouse have strongly supported this view. However, in the chick, we have observed a dual origin of LECs from veins and from mesodermal lymphangioblasts. Additionally, in murine embryos we have detected mesenchymal cells that co-express LEC markers and the pan-leukocyte marker CD45. Here, we have characterized the mesoderm of murine embryos with LEC markers Prox1, Lyve-1 and LA102 in combination with macrophage markers CD11b and F4/80.

**Results:**

We observed cells co-expressing both types of markers (e.g. Prox1 – Lyve-1 – F4/80 triple-positive) located in the mesoderm, immediately adjacent to, and within lymph vessels. Our proliferation studies with Ki-67 antibodies showed high proliferative capacities of both the Lyve-1-positive LECs of lymph sacs/lymphatic sprouts and the Lyve-1-positive mesenchymal cells.

**Conclusion:**

Our data argue for a dual origin of LECs in the mouse, although the primary source of embryonic LECs may reside in specific embryonic veins and mesenchymal lymphangioblasts integrated secondarily into lymph vessels. The impact of a dual source of LECs for ontogenetic, phylogenetic and pathological lymphangiogenesis is discussed.

## Background

The important physiological and pathophysiological roles of the lymphatic vascular system for fluid homeostasis, immune surveillance, inflammation and tumour metastasis justify intensive studies of this hardly visible portion of the vascular system [1,2]. Insufficient development of lymph vessels becomes immediately apparent as lymph oedema, which mostly affects the legs and the genital region of patients. Primary lymph oedema (Nonne-Milroy Syndrome) is caused by mutations in the tyrosine kinase domain of the Vascular Endothelial Growth Factor Receptor-3 (VEGFR-3) gene on 5q35.3 [3,4]. Kaposi's sarcoma probably represents a form of lymphatic endothelial cell (LEC) hyperplasia [5], but circulating precursor cells may also be involved [6]. However, it is still not clear whether lymphangioma, which is found in 1.2 – 2.8‰ of infants [7], is due to hyperplasia of LECs or structural malformations of lymph vessels [8]. These uncertainties with respect to the pathobiology of lymph vessels are based on the fact that the mechanisms of normal embryonic lymphangiogenesis and the origin of LECs are not sufficiently well understood.

Following the identification of specific markers for LECs, our knowledge of the structure and function of lymph vessels and the molecular equipment of LECs has increased enormously in recent years [9,10]. Nevertheless, the embryonic origin of the lymphatic vascular system has been discussed controversially for more than a hundred years, and is still open for discussion. The two main theories are the 'centrifugal' and 'centripetal' theory. The first was set up by Sabin [11,12] and Lewis [13] and proposes a venous origin of the lymph sacs (which are the first clear morphological signs of lymph vessel development), with subsequent sprouting of lymph vessels into all tissues and organs of the body. The second was set up by Huntington and McClure [14] and proposes formation of lymphatic vessels from mesenchymal 'lymphatic clefts', which, nowadays, are called lymphangioblasts. Several recent studies clearly show development of LECs from the venous system in the murine embryo [15-17]. However, an intermediate position favouring a dual origin from embryonic veins and mesenchymal lymphangioblasts was defined by studies on avian embryos and Xenopus tadpoles [18-20]. Migration of mesenchymal lymphangioblasts, which are originally located in the venous system and delaminate from the endothelium, has been observed in fish [21]. Additionally, our previous studies have demonstrated the existence of mesenchymal cells which co-express leukocyte (CD45) and lymphendothelial markers (Prox1, Lyve-1) in mouse embryos [19]. Recent studies on pathological lymphangiogenesis in adult mice have provided evidence of a role of circulating endothelial progenitor cells (CEPCs) and macrophages (CD11b and F4/80 positive) in this process [22-24]. Evidence of CEPCs in humans has been provided in studies on post-transplantation Kaposi's sarcoma [6] and kidney graft rejection [25].

The detection of cells, which co-express macrophage and lymphendothelial markers in the adult mouse, prompted us to investigate such cells in the murine embryo. We used antibodies against CD31/PECAM-1, a pan-endothelial marker, in combination with LEC-specific markers: Prox1, a homeobox transcription factor, Lyve-1, a hyaluronan receptor, and LA102, a recently defined new epitope on LECs [26]. Double and triple staining were then performed with macrophage markers CD11b and F4/80. These studies, in combination with the proliferation marker Ki-67, provide evidence for the existence of actively dividing mesenchymal cells, which co-express macrophage and lymphendothelial markers in early mouse embryos. The mesenchymal localization of the cells in murine embryos suggests an additional source of LECs, which may integrate into lymph vessels that have primarily originated and sprouted from embryonic veins. The existence of highly mobile (macrophage-like) cells with LEC characteristics seems to be relevant for pathological lymphangiogenesis and inflammation-associated organ rejection [reviews see: [27,28]].

## Results

### Cells with pan-endothelial, lymphendothelial and macrophage characteristics

**Figure 1:**
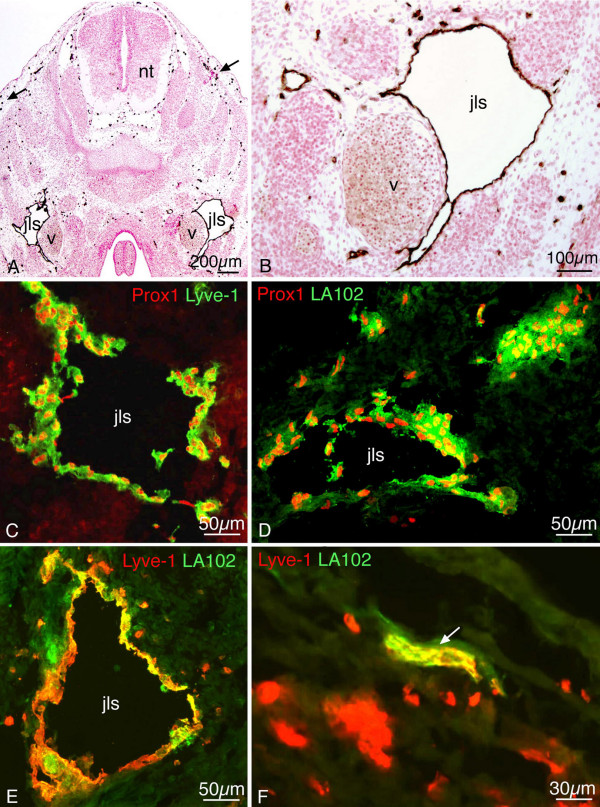
**Jugular lymph sacs of murine embryos**. Lyve-1 staining of paraffin sections (A, B) and double staining of cryo-sections (C-F) of ED 13.5 (A-C, E, F) and 12.5 (D) mice. A, B) The jugular lymph sacs (jls) are Lyve-1^+ ^and located dorso-laterally of the cardinal vein (v). Many scattered Lyve-1^+ ^cells are located superficially in the developing dermis (arrows). nt, neural tube. B) Higher magnification of A showing JLS in close proximity to the cardinal vein. C) Lymph sacs express Prox1 (red) and Lyve-1 (green). D) Prox1 (red) and LA102 (green). E) Lyve-1 (red) and LA102 (green). Note double positive LECs, although staining of lymph sac LECs with LA102 is heterogeneous. F) Lymphatic vessel (arrow) in the dermis is double positive for Lyve-1 and LA102 (yellow).

During early stages of lymphendothelial commitment, the LEC markers Prox1 and Lyve-1 are expressed by cells of the cardinal veins of the mouse on approximately embryonic day (ED) 9.5 [17]. These cells are also positive for CD31/PECAM-1, a pan-endothelial marker, which is strongly expressed in blood endothelial cells (BECs) and more weakly in LECs [29,30]. The precursor cells give rise to lymph sacs which, in the jugular region, are located in the angle between anterior and posterior cardinal veins [15], as can be demonstrated by staining with anti-Lyve-1 antibodies (Fig. 1). However, the lymph sacs are not the only structures which are Lyve-1-positive. There are large numbers of scattered cells, preferentially located in the loose mesenchyme (Fig. 1A). On ED 13.5 the lymphatic vascular system of murine embryos is made up of a deep and a superficial part. The deep part consists of the lymph sacs located alongside the jugular segment of the cardinal veins (Fig. 1A,B). The superficial lymphatics are located in the dermis. They are connected to the deep system by lymphatic vessels, which are located in the mesenchyme underneath the skeletal elements of the shoulder girdle. Additionally, there are large numbers of scattered Lyve-1-positive cells in the loose mesenchyme of the dermis, the primitive meninges and the parapharyngeal region. The neural tube, skeletal elements and muscles do not contain such cells.

Our double staining protocol confirms that LECs of the jugular lymph sacs are Prox1^+ ^and Lyve-1^+ ^(Fig. 1C). The new lymphendothelial marker LA102, a monoclonal antibody recognizing a 25 – 27 kDa protein, specifically reacts with lymphatic vessels of the adult mouse, except for those of the thoracic duct and the marginal sinus of lymph nodes, but not with any blood vessels [26]. We investigated the expression of LA102 during early embryonic lymphangiogenesis of ED 11.5 – 13.5 mice and performed double staining with either Prox1 or Lyve-1. On ED 12.5 and ED 13.5, we observed that LECs in the jugular lymph sacs were double positive for Prox1 and LA102 (Fig. 1D) as well as Lyve-1 and LA102 (Fig. 1E). The same results hold true for the superficial lymphatics of the dermis (Fig. 1F). However, in the mediastinal region, we found some lymphatic vessels which expressed only Lyve-1, while others demonstrated Lyve-1 and LA102 co-expression (data not shown).

**Figure 2:**
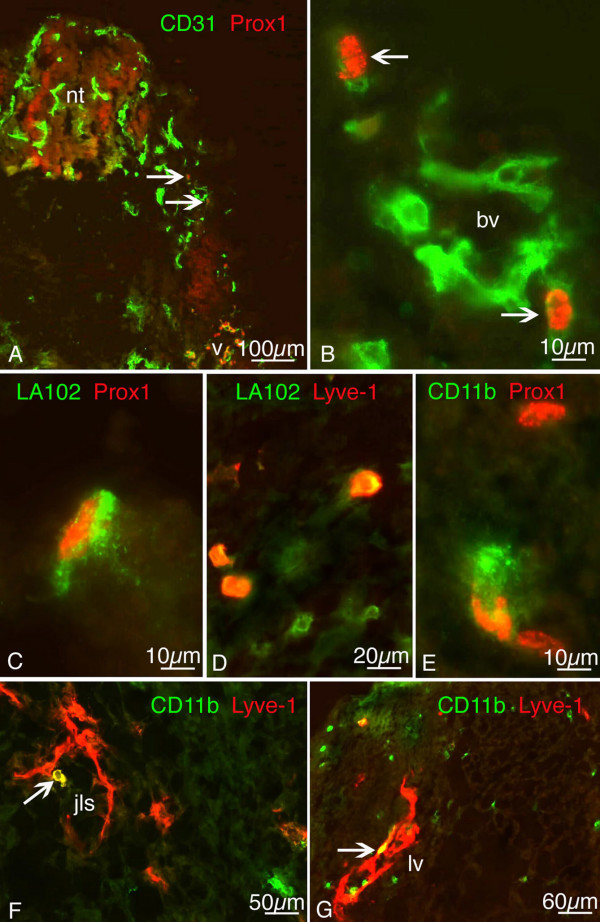
**Double staining of scattered mesodermal cells with pan-endothelial, lymphendothelial and macrophage markers of ED 10 (A, B), ED 12.5 (D, E) and ED 13.5 (C, F, G) mouse embryos**. A) Two mesenchymal cells of the dermatome (arrows) which are located at a great distance from the cardinal vein (v) express CD31 (green) and Prox1 (red). nt, neural tube. B) Higher magnification of A). Note weak CD31 expression of Prox1^+ ^mesenchymal cells (arrows) as compared to blood vessels (bv). C) Cells double positive for lymphendothelial markers LA102 (green) and Prox1 (red), and D) LA102 (green) and Lyve-1 (red). E) Cells double positive for macrophage marker CD11b (green) and lymphendothelial marker Prox1 (red). F) Some cells co-express (yellow) CD11b (green) and Lyve-1 (red) in the mesenchyme adjacent to the JLS, and G) in the dermis, where a cell positive for both markers (arrow) seems to be integrated into a lymph vessel (lv).

The data show that all three markers, Prox1, Lyve-1 and LA102 are expressed by LECs of lymph sacs and lymph vessels, although each is also expressed by some non-endothelial cell types. In order to characterize the scattered mesenchymal Lyve-1^+ ^cells shown in Figure 1A, we used double and triple staining with lymph/endothelial and macrophage markers. In our previous studies, we showed that some mesenchymal Lyve-1^+ ^cells co-expressed Prox1 [19]. Here, we detected a small number of Prox1^+^/CD31^+ ^mesenchymal cells in the dermatomes on ED 10 (Fig. 2A,B). CD31 expression in these cells, which we regard as lymphangioblasts, is lower than in BECs. Co-staining with LA102 and Prox1 antibodies revealed that some scattered cells in the mesenchyme are double positive (Fig. 2C). On ED 12.5 we observed single cells in the mesenchyme surrounding the neural tube which co-expressed Lyve-1 and LA102, but there were also cells only positive for LA102 (Fig. 2D). It has been reported that CD11b^+ ^macrophages may contribute to pathological lymphangiogenesis in the cornea of adult mice [22]. We detected a small number of Prox1^+^/CD11b^+ ^cells in the mesenchyme of ED 12.5 mice (Fig. 2E). LECs of the jugular lymph sacs on ED 13.5 were CD11b-negative, but a few Lyve-1^+^/CD11b^+ ^cells were located immediately adjacent to the lymph sacs (Fig. 2F), and appeared to be integrated into the superficial dermal lymph vessels (Fig. 2G).

**Figure 3:**
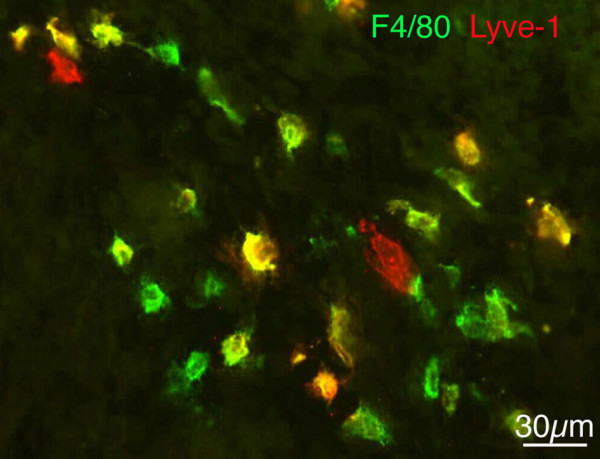
**F4/80 (green) and Lyve-1 (red) staining of ED 13.5 mice**. Many of the cells in the dermis are double positive (yellow), but some only express F4/80 or Lyve-1.

**Figure 4:**
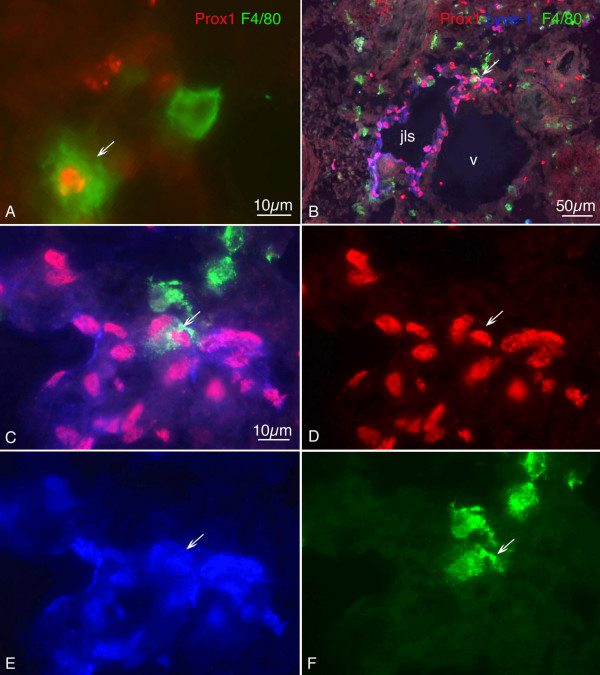
**Double and triple staining of ED 11.5 (A) and ED 13.5 (B – F) mouse embryos with Prox1 (red), Lyve-1 (blue), and F4/80 (green)**. A) Cell showing co-expression of Prox1 and F4/80 (arrow), whereas another cell is only F4/80 positive. B) Overview showing the jugular lymph sac (jls) and the cardinal vein (v). Prox1 and Lyve-1 are co-expressed in LECs of the jls. Arrow indicates integration of F4/80^+ ^cell into the jls. C) Higher magnification of B) showing triple positive cells of the JLS. D) Prox1, E) Lyve-1, F) F4/80.

**Figure 5:**
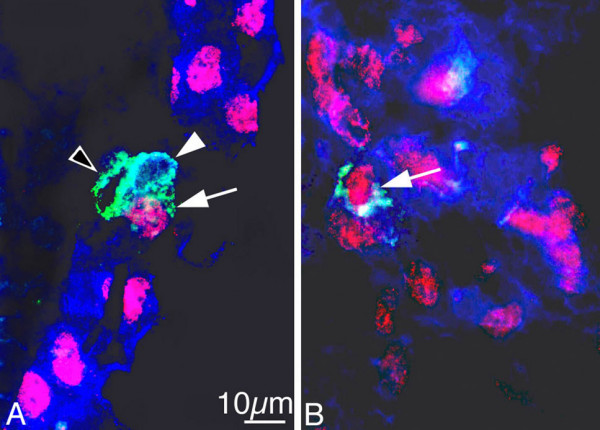
**Triple staining of ED 13.5 mouse embryos with Prox1 (red), Lyve-1 (blue), and F4/80 (green)**. A, B) Triple positive cell integrated in a lymphatic vessel (arrow in A and B). A) Cell showing co-expression of Lyve-1 and F4/80 (white arrowhead), whereas another cell is only F4/80 positive (open arrowhead).

We then used the macrophage marker F4/80 for further characterisation of mesenchymal Lyve-1^+ ^cells. A large number of scattered cells of the dermatomes, mediastinum and pimitive meninges co-expressed Lyve-1 and F4/80 in ED 13.5 embryos and some cells expressed only Lyve-1 or F4/80 (Fig. 3). There was a distinct difference between the frequencies of CD11b^+ ^and F4/80^+ ^cells in the mesenchyme. Many more F4/80^+ ^cells could be found, and many of these were Lyve-1^+^. We, therefore, extended our studies by applying three markers: Prox1, Lyve-1 and F4/80. We found cells in the developing lymph sacs positive for all three markers in ED 13.5 mice (Fig. 4A–F). More peripherally, in developing lymph vessels, we also found a small number of cells positive for all three markers (Fig. 5A,B). In very rare cases, we could observe various marker combinations in adjacent cells of lymph vessels. Figure 5A shows such an example with 3 cells: one positive only for F4/80, one expressing F4/80 and Lyve-1, and a third positive for F4/80, Lyve-1 and Prox1. The fact that two LEC markers, Prox1 and Lyve-1, can be found in F4/80^+ ^cells, which appear to be integrated into lymph vessels, is a strong indication for a mesenchymal source of LECs in murine embryos.

### Proliferation studies

**Figure 6:**
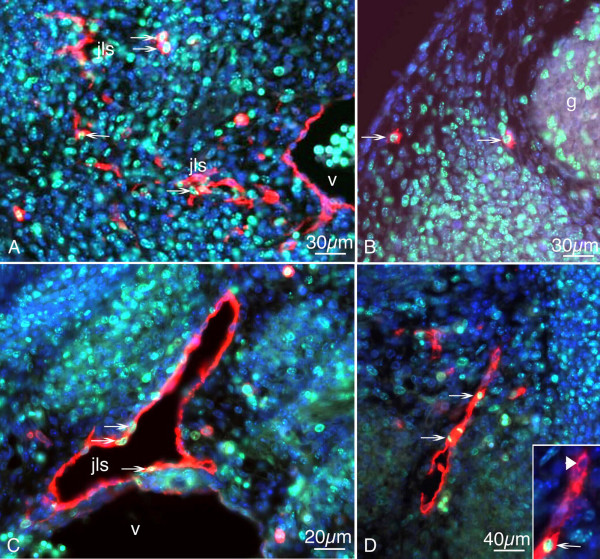
**Paraffin sections of mouse embryos stained with antibodies against Ki-67 (green) and Lyve-1 (red)**. Counter staining was performed with DAPI (blue). A) On ED 11.5, there are proliferating LECs in the developing jugular lymph sacs (jls) (arrows), but no double positive cells in the cardinal vein (v). B) Two mesenchymal cells positive for the markers located in the dermatome (arrows). g, spinal ganglion. C) JLS of ED 12.5 mice with LECs (arrows) expressing Ki-67 and Lyve-1. D) Sprout from the JLS containing proliferating cells (arrows). Inset: Higher magnification showing Ki-67-positive (arrow) and negative (arrowhead) LECs.

**Figure 7:**
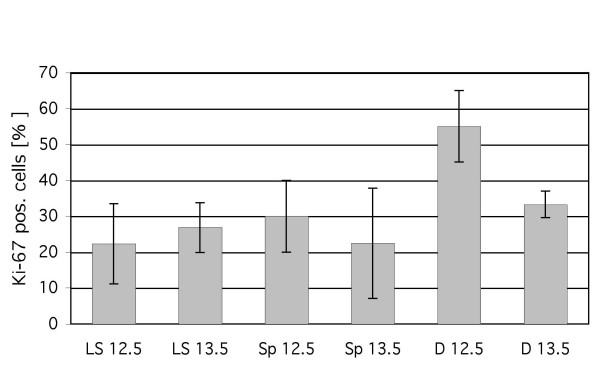
**Proliferation studies of Lyve-1^+ ^cells with Ki-67 antibodies in ED 12.5 and ED 13.5 mice**. For each ED, at least 5 sections of at least 3 mice were evaluated. The total number of cells counted was: 197 in lymph sacs (LS) on ED 12.5; 406 in lymph sacs (LS) on ED 13.5; 45 in sprouts from lymph sacs (Sp) on ED 12.5; 75 in sprouts from lymph sacs (Sp) on ED 13.5; 43 in scattered cells in the dermatomes (D) on ED 12.5 and 40 in scattered cells in the dermatomes (D) on ED13.5. Mean and standard deviation are shown. The value for D 12.5 is significantly higher than all others.

Ki-67 is a well-established proliferation marker and is increasingly expressed in the nucleus in all active phases of the cell cycle, but not in G_0 _phase [31]. To extend our studies on the source of LECs, we performed double staining with anti-Ki-67 and anti-Lyve-1 antibodies of ED 11.5 – 13.5 mice. Nuclei were counter stained with 4',6-diamidino-2-phenylindol (DAPI). Double positive LECs could be detected in regions of active lymphangiogenesis: the early jugular veins, the jugular lymph sacs, which extended sprouts into dorso-lateral directions, the mediastinum and the dermis (Fig. 6). Mouse embryos on ED 11.5 exhibited very few cells in the cardinal veins positive for both markers, whereas LECs of the developing jugular lymph sacs as well as mesenchymal cells in the dermis co-expressed Ki-67 and Lyve-1 at high rates (Fig. 6A,B). The dermatomes of ED 12.5 and 13.5 mice displayed scattered Lyve-1^+ ^cells, which also showed proliferating characteristics (55% and 33%, respectively; Fig. 7). However, the total number of Lyve-1^+ ^cells in the dermatomes was low compared with the number of lymph sac ECs. In the jugular lymph sacs (Fig. 6C) and in sprouts derived from them (Fig. 6D) the Ki-67 labelling rate was 22 – 30% (Fig. 7). The data show that a greater number of Lyve-1^+ ^cells are present in the lymph sacs than in the dermatomes, but the latter proliferate at equal or, in ED 12.5 embryos, even higher rates.

## Discussion

### Embryonic lymphangiogenesis

Our data show that there is a cell type in early mouse development, which combines lymphendothelial (Lyve-1^+^, Prox1^+^) and macrophage (F4/80^+^) characteristics. Also, there are cells that express Lyve-1 and F4/80, as well as cells that have only one of these markers. The triple-positive cells appear to be integrated into the lining of early lymph vessels, and, in sum, the data are in favour of an origin of LECs from mesenchymal, macrophage-like cells. Although these cells may contribute only to the minor part of the lymphovascular system, their existence and possible contribution to pathological lymphangiogenesis should not be neglected.

Recent data by Srinivasan et al. [15] have provided clear evidence that the major part of the lymphovascular system of mice is of venous origin. The jugular segment of the cardinal veins is the source of the jugular lymph sacs, which then grow into peripheral parts of the body. Prox1-expressing venous ECs are the source of the jugular lymph sacs in the anterior region of the embryo. The posterior cardinal veins give rise to the posterior lymph sacs, which are the source of the lymphatics in the mesenteries and the posterior organs. The earliest Prox1-positive ECs are located in the jugular segment of the cardinal veins of ED 9.75 mice. A third place of origin of lymphatics is the orbital region [15]. Loss of Prox1 expression abolishes development of lymphatics [17]. Regular development of veins is another prerequisite for the generation of LECs. The absence of Coup-TFII, an orphan nuclear receptor transcription factor, causes embryonic death around ED 10, abnormal expression of arterial marker genes in the veins, and loss of jugular lymph sacs [15,32-36]. A contribution of hematopoietic cells to the lymphatics does not seem to exist. *Runx1*-null mice, which do not develop definitive hematopoietic cell [37], have normal jugular lymph sacs, and descendents of hematopoietic stem cells are not detectable in the mouse carrying a reporter gene under the control of the *Runx1 *promoter [15]. In all, these data show that the venous system is the main source of the embryonic lymphovascular system in mice.

Nevertheless, even the highly detailed studies on the venous origin of embryonic LECs cannot rule out the possibility of a minor contribution of mesenchymal cells to the lymphatics [15,17]. In both of the studies cited, scattered Prox1-positive cells were observed along the anteroposterior axis of the body wall of early embryos – at great distances from the developing lymph sacs. In the transgenic mice studied by Srinivasan et al. [15] some cells were detected, which did not fit into the anticipated pattern of the venous origin of LECs. Thus, there are some observations that argue for an additional source of LECs. The number of LECs derived from macrophage-like, mesenchymal precursors may be low, but that does not necessarily rule out functional importance. This is supported by studies on *Syk *and *Slp-76 *knockout mice. These adapter proteins mediate outside-in integrin signalling [38,39]. Mouse embryos lacking Syk and Slp-76 functions develop abnormal blood-lymph-endothelial connections, and Slp-76 deficient cells confer this phenotype on chimeric embryos [40,41]. *Syk *and *Slp-76 *are expressed in hematopoietic cells, but not in ECs. The incorporation of defective cells into abnormally fused lymph vessels and the rescue of the phenotype by GFPSlp-67-expressing circulating cells indicate a hematopoietic origin of LECs [40]. Normal *Syk *and *Slp-76 *functions may be required in early stages of development, when hemangioblasts divide into hematopoietic and endothelial lineages. *Syk *and *Slp-76 *are expressed by platelets and eosinophils, but not macrophages or dendritic cells [40].

Incorporation of macrophages into lymph vessels has been indicated by studies on pathological lymphangiogenesis [22-24]. Our studies strongly suggest that similar mechanisms are found in embryonic lymphangiogenesis. In the adult, macrophages originate from circulating monocytes, but, in the embryo, their early development in the mesoderm is independent of the hematopoietic system. They can be isolated from the epiblast before gastrulation. So, they are present before the development of hematopoietic cells in blood islands [42]. After gastrulation, macrophages are found in all embryonic compartments, which is also evident in the studies presented here. Mesenchymal macrophages can give rise to stationary cells, which is best documented in the central nervous system (CNS) where they form the microglia [43], which has self-renewal potential. Our studies provide initial evidence for the incorporation of macrophage-like cells into the lining of lymph vessels during normal development.

There are large numbers of Lyve-1^+ ^single cells in the mesenchyme of murine embryos, which are highly proliferative and obviously represent a heterogeneous cell population. Many of the cells express the pan-leukocyte marker CD45 [19]. Here, we show that some of the cells co-express other LEC markers such as Prox1 and LA102. Co-localization of Prox1 and CD31/PECAM-1 in scattered cells in the dermatomes of ED 10 mouse embryos is a strong indicator of a lymphangioblastic cell population. Prox1 is the most reliable marker that distinguishes between LECs and BECs in healthy and diseased tissues [29,30], and CD31 is a highly reliable pan-endothelial marker. In birds, similar cells are present, and grafting of quail dermatomes into chick embryos has demonstrated the lymphangiogenic potential of this tissue [20]. Many of the Lyve-1^+ ^single cells in the mesenchyme express F4/80, but only some are CD11b^+^. The F4/80 antibody recognises an extra-cellular membrane molecule, highly restricted to mature macrophage subpopulations. The antigen is not present on granulocytes, lymphocytes, mast cells, platelets, endothelial cells and blood monocytes [44]. CD11b is found on 'classical' macrophages such as alveolar and peritoneal macrophages, but not on dendritic cells [45]. We detected a small number of Prox1-Lyve-1-F4/80 triple-positive cells, which were obviously integrated into the lining of lymph vessels. Although this is a rare event, it demonstrates heterogeneity of LECs, probably due to heterogeneity of lymphangiogenic mechanisms, which may be relevant not only during embryonic lymphangiogenesis, but also in phylogeny and during pathological lymphangiogenesis.

### Phylogenetic lymphangiogenesis

Development of LECs from macrophage-like progenitors might reflect an ancestral mechanism. This assumption is based on the observation that insects, which do not possess ECs [46], have macrophages that express ancestors of typical angiogenesis-associated molecules of vertebrates [47-49]. The tubular heart (also called dorsal vessel) of insects is lined by contractile muscle cells, but not ECs [46]. In *Drosophila *supply with oxygen is accomplished by a tracheal system. Red blood cells are not present and the so-called hemolymphatic system fulfils functions of a typical lymphatic system. It has, therefore, been speculated that the lymphovascular system evolved before the blood vascular system, although during ontogeny the latter develops first [50]. Three types of immune cells are found in *Drosophila*: plasmatocytes, crystal cells and lamellocytes [51]. Plasmatocytes, which are capable of phagocytosis, are comparable to the monocyte/macrophage lineage of vertebrates. They possess a single ancestor for the VEGF (Vascular Endothelial Growth Factor) and PDGF (Platelet-derived Growth Factor) receptors found in vertebrates. This receptor, called PVR, directs immune cell migration [48,49,52]. The VEGF-PDGF-system is indispensable for the development of vessels in vertebrates (ECs, pericytes, smooth muscle cells), and it is tempting to speculate that vascular wall cells are derived from macrophage-like cells during phylogeny, and some LECs still do so during ontogeny.

### Pathological lymphangiogenesis

Lymphangiogenesis in embryos and adult organisms have many mechanisms in common, but they may also differ to some extent. Lymphangiogenesis in the adult takes place in the proliferation phase of the endometrium [53]. Pathological lymphangiogenesis can be observed in inflammatory diseases such as Crohn's disease and ulcerative colitis [54], in and around tumours that express the lymphangiogenic growth factors VEGF-C and -D [55-57], and during wound healing, where it occurs after blood vessels have formed [58]. In these scenarios, new lymphatics are mostly formed from pre-existing ones, however, exceptions have been observed. One of these exceptions is the healing of dermal wounds in the mouse tail [59]. In this model, LECs do not sprout from lymph vessels, but migrate as single cells from distal to proximal parts of the tail and organize into vessels in the direction of interstitial fluid flow. It is tempting to speculate that the dermis of adult organisms still contains lymphendothelial precursor cells, which is in line with our observation of lymphangioblasts in the early dermatomes of murine and avian embryos [2,19].

Macrophages may support pathological lymphangiogenesis in two ways: either by transdifferentiation and direct incorporation into the endothelial layer or by stimulating division of pre-existing local LECs [22,23,60]. Thereby, VEGF-C seems to have a dual role as inducer of LEC proliferation and chemoattractant of macrophages [61]. In diabetes, wound healing is impaired, due to decreased numbers of macrophages (F4/80^+^, Lyve-1^+^, podoplanin^+^) and lymph vessels [62]. A pool of macrophages expressing VEGFR-3, CD45 and CD11b has been found in the conjunctiva of normal and inflamed murine eyes [63]. However, further studies are needed to clarify whether their incorporation into lymph vessels is permanent or represents some kind of lymphendothelial mimicry [24].

Evidence for circulating cells with lymphendothelial characteristics has been found in human diseases. In patients with gender-mismatched renal transplants and inflammation-associated transplant rejection, Kerjaschki et al. [25] observed lymphangiogenesis with Ki-67 labelling rates of 2.3% of LECs, and incorporation of circulating recipient-derived cells into kidney lymph vessels in the range of 2.7 – 7%. The Ki-67 labelling rates are clearly lower than those we observed in embryonic LECs (in the mouse), whereas the incorporation of circulating progenitor cells into the lining of lymph vessels appears to be relatively high in the adult human. The second disease, which shows involvement of circulating lymphatic progenitor cells, is Kaposi's sarcoma (KS). The cell of origin in KS has long been a subject of debate. Mesenchymal cells, smooth muscle cells, BECs, LECs, Schwann cells and fibroblasts have been discussed. An endothelial origin has then been favoured, but it has remained unclear whether these are BECs or LECs [64,65]. Studies on the expression of lymphendothelial markers VEGFR-3, podoplanin and Lyve-1, and studies showing the mitogenic effect of VEGF-C seem to prove that KS is derived from LECs [5]. However, examination of biopsies from KS skin lesions, which developed after transplantation in immuno-suppressed recipients of kidneys, showed that a high proportion of KS cells were of donor (kidney) origin [6]. These findings suggest that KS cells are derived from highly mobile cells. It is probable that these are macrophage-like, which originated in the donor organ. Cells with macrophage and endothelial characteristics have been isolated from peripheral blood of KS patients [66].

## Conclusion

In summary, we describe mesenchymal cells with macrophage and lymphendothelial characteristics in murine embryos. These cells seem to participate in normal lymphangiogenesis and might represent a relict of the phylogenetic development of lymph vessels from immune cells. Similar cells may take part in pathological lymphangiogenesis in the mouse and in human beings, and may be the cell of origin in Kaposi's sarcoma.

## Methods

### Animals

NMRI and C57BL/6 wild type mouse embryos from ED 10 to 13.5 were studied. Embryos were removed from the uterus and remained either unfixed, or were fixed in 4% paraformaldehyde (PFA), or in 100% ethanol containing 3% acetic acid. We did not observe any differences between the two strains.

### Immunohistochemistry

Mouse embryos were embedded in paraffin and sectioned into 8 μm slices with a microtome (Leica, Bensheim, Germany) and placed onto slides. Sections were stained with rabbit-anti-mouse Lyve-1 antibodies (Regeneron, Tarrytown, NY). The secondary antibody was peroxidase-conjugated goat-anti-rabbit IgG (Sigma-Aldrich, Taufkirchen, Germany). Diaminobenzidine was used as chromogen. Sections were counter stained with nuclear fast red.

### Proliferation studies

Paraffin sections were stained with rabbit-anti-mouse Lyve-1 antibodies [67,68] and Ki-67 (Dako, Hamburg, Germany). The Ki-67-antigen was used with citrate buffer in the microwave oven for antigen retrieval. The secondary antibodies, Alexa 488-conjugated goat-anti-rat IgG, and Alexa 594-conjugated goat-anti-rabbit IgG (Molecular Probes, Eugene, US), were applied at a dilution of 1:200, for 1 hour (h). Sections were counter-stained with DAPI.

### Immunofluorescence

Embryos of various developmental stages remained unfixed or were fixed in 4% PFA for 20 minutes. They were rinsed in phosphate-buffered saline (PBS), transferred to 5% and 15% sucrose in PBS, and embedded in Tissue Freeze Medium (Sakura Finetek Europe, NL). Cryosections of 20 μm thickness were prepared. Non-specific binding of antibodies was blocked by incubation with 1% bovine serum albumin (BSA) for 1 h before incubation with the primary antisera. The following primary antibodies were used in this study for double and triple staining: rabbit-anti-mouse LYVE-1 (Regeneron, Tarrytown, NY), rat-anti-mouse CD31/PECAM-1 (1:50; BD Pharmingen, San Diego, US), rabbit-anti-human Prox1 (1:750; ReliaTech, Braunschweig, Germany), rat-anti-mouse Ki-67 (1:50; Dako, Hamburg, Germany), rat-anti-mouse CD11b (1:50; BD Pharmingen, San Diego, US), rat-anti-mouse F4/80 (1:100; Hycult Biotechnology, Uden, Netherlands), and rat-anti-mouse LA102 (pure supernatant, T. Ezaki, Japan). The sections were incubated with the primary antibodies for 1 h, except for Prox1, which was incubated overnight. In the negative controls, the primary antibody was omitted. Typical controls have been published [19]. After having been rinsed, the secondary Alexa 488-conjugated goat-anti-rat IgG and Alexa 594-conjugated goat-anti-rabbit IgG (Molecular Probes, Eugene, US) were applied at a dilution of 1:200. Alexa 350-conjugated goat-anti-rabbit IgG (Molecular Probes) was applied at 1:50 for 1 h. After having been rinsed, the sections were mounted under cover slips with Fluoromount-G (Southern Biotechnology Associates, Birmingham, GB). They were then viewed with the DM5000B epifluorescence microscope (Leica, Bensheim, Germany) and Zeiss Axioplan 2 LSM 510 (Zeiss, Göttingen, Germany).

## Authors' contributions

KB carried out immunostaining and was involved in the analysis of data and preparation of the manuscript. TE performed immunostaining with lymphendothelial marker LA102. JW conceived the study and participated in its design, data analysis and manuscript preparation.
